# A Meta-Analysis to Determine the Impact of Restaurant Menu Labeling on Calories and Nutrients (Ordered or Consumed) in U.S. Adults

**DOI:** 10.3390/nu9101088

**Published:** 2017-09-30

**Authors:** Thaisa M. Cantu-Jungles, Lacey A. McCormack, James E. Slaven, Maribeth Slebodnik, Heather A. Eicher-Miller

**Affiliations:** 1 Department of Food Science, Purdue University, West Lafayette, IN 47907, USA; thaisamoro@gmail.com; 2 Department of Health and Nutritional Sciences, South Dakota State University, Brookings, SD 57007, USA; Lacey.McCormack@sdstate.edu; 3 Department of Biostatistics, Indiana University School of Medicine, Indianapolis, IN 46202, USA; jslaven@iu.edu; 4 Arizona Health Sciences Library, University of Arizona, Tucson, AZ 85724, USA; slebodnik@email.arizona.edu; 5 Department of Nutrition Science, Purdue University, West Lafayette, IN 47907, USA

**Keywords:** menu labeling, food labeling, nutritional labeling, meta-analysis, energy, calories, nutrients, adults, point-of-purchase

## Abstract

A systematic review and meta-analysis determined the effect of restaurant menu labeling on calories and nutrients chosen in laboratory and away-from-home settings in U.S. adults. Cochrane-based criteria adherent, peer-reviewed study designs conducted and published in the English language from 1950 to 2014 were collected in 2015, analyzed in 2016, and used to evaluate the effect of nutrition labeling on calories and nutrients ordered or consumed. Before and after menu labeling outcomes were used to determine weighted mean differences in calories, saturated fat, total fat, carbohydrate, and sodium ordered/consumed which were pooled across studies using random effects modeling. Stratified analysis for laboratory and away-from-home settings were also completed. Menu labeling resulted in no significant change in reported calories ordered/consumed in studies with full criteria adherence, nor the 14 studies analyzed with ≤1 unmet criteria, nor for change in total ordered carbohydrate, fat, and saturated fat (three studies) or ordered or consumed sodium (four studies). A significant reduction of 115.2 calories ordered/consumed in laboratory settings was determined when analyses were stratified by study setting. Menu labeling away-from-home did not result in change in quantity or quality, specifically for carbohydrates, total fat, saturated fat, or sodium, of calories consumed among U.S. adults.

## 1. Introduction

Excess adiposity contributing to the “obesity epidemic” is a public health concern in the United States [1,2,3,4]. In 2011–2012, more than two-thirds (approximately 68.5%) of adults were overweight or obese (defined as a body mass index ≥25) [5], an approximate 12% increase since 1988–1994 [6]. The financial burden associated with the U.S. overweight/obesity prevalence combined is estimated at approximately 5–10% of total healthcare costs annually [7]. Obesity is also associated with an increased risk of morbidity for several health conditions, including hypertension [8,9,10,11,12], type 2 diabetes [13,14,15,16], and cardiovascular disease [17,18,19,20] among U.S. adults. Many factors may contribute to the prevalence of overweight and obesity, including environment, genetics, and behavior choices, such as physical activity and diet including excess caloric intake [1,20,21,22]. Food consumed away from home, including foods from table-service restaurants, cafeterias, taverns, and fast food [23], may be one such contributing factor.

U.S. consumer spending on food away-from-home has increased since the mid-1990s and is expected to continue to increase by approximately 18% at full-service restaurants and 6% at fast-food restaurants between 2000 and 2020, indicating the importance of away-from-home foods to U.S. dietary intake [24]. The availability of fruits and vegetables, whole grains, and low-fat milk in restaurants is limited according to an evaluation of the nutrition environment in the away-from-home setting [25]. Foods purchased away from home tend to have high energy per nutrient density when compared with foods consumed at home. Main and side dishes at sit down and fast-food restaurant chains consistently contained high amounts of fat, saturated fat and sodium, and low amounts of fiber and fruits/vegetables [26]. An evaluation of approximately 3500 meals at 34 of the top 50 U.S. restaurant chains showed that 91% of meals did not meet dietary standards created by the National Restaurant Association [27]. Thus, although food prepared away from home accounts for a significant share of U.S. food consumption, it may exert a disproportionately larger influence on the intake of nutrients of public health concern compared with food prepared at home [28,29,30]. 

Several studies have proposed that food prepared away from home may be a contributor to overweight and obesity in the U.S. [29,31,32,33,34,35]. Consumers were poor estimators of the calories contained in restaurant food items [36,37,38] and the consumption of food away from home was positively associated with caloric intake [31]. The lack of nutrition information in restaurant establishments was given as a reason for consumer difficulty in making healthy choices, suggesting a need for menu labeling [31]. A variety of factors may influence away-from-home food purchases, but it is plausible that this type of nutrition labeling on menus in restaurant establishments would be used by consumers when a third of the population use nutrition information on packaged foods [39]. In response, the Obesity Working Group advised the Food and Drug Administration (FDA) to encourage restaurants to provide nutrition information to help consumers make healthy choices [40] and following this, a population-based public health initiative targeting food prepared away from home was implemented to help ameliorate the rising rates of overweight and obesity. Section 4205 of the Patient Protection and Affordability Care Act passed in 2010 required that chain restaurants and similar retail food establishments with 20 or more sites post calorie information for menu items at the point-of-purchase [41] to help consumers make healthy choices when eating food prepared away from home. 

Such far-reaching policy has the potential to effect overall U.S. away-from-home dietary intake. Several previous systematic reviews and meta-analyses have been completed to evaluate the impact of menu labeling on calories chosen in an away-from-home setting [42,43,44,45,46] but evaluation regarding nutrient intakes, specifically carbohydrates, total fat, saturated fat, and sodium, has not been included. Estimation of the purchase or intake of these nutrients in the overall U.S. dietary context of excessive calorie intake, not including other populations [43,46], and the special designation of saturated fat and sodium intake as nutrients of concern, is critical to determine how menu labeling may be influencing not only the quantity of U.S. caloric energy but also indicators in order to begin to quantify the quality of energy from foods consumed away from home. Adults primarily make away-from-home purchasing decisions, thus the previous systematic reviews of Long et al. [42], Swartz et al. [44], and Sinclair et al. [43] including non-adults, do not provide clear information on adult populations. Finally, evaluation of purchase intentions only [45], may not reflect actual ordering/consumption behavior among U.S. adults. Therefore, the novel hypothesis of this study was that restaurant menu labeling would not affect caloric choice or intake but would alter carbohydrate, total fat, saturated fat, and sodium choice or intake compared with before menu labeling or compared with a control group, in an away-from-home setting in the U.S. adult population. The hypothesis is based on the previous literature showing null or minimal differences in calorie choice and attempts to fill the gap of knowledge indicating whether menu labeling may impact chosen or consumed nutrients. Secondly, the effect of menu labeling in a natural away-from-home setting or in a lab environment was hypothesized to make a difference in the efficacy of menu labeling as an intervention to alter caloric choice or intake among U.S. adults. Data were handled to prevent the interference of interventions other than quantitative nutritional labeling (education, taxation, etc.) and a special emphasis on high quality study designs was implemented in order to inform future implementation of this policy intended to support the prevention and reduction of obesity and other chronic diseases.

## 2. Materials and Methods

### 2.1. Study Identification and Selection

This meta-analysis was conducted according to the Preferred Reporting Items for Systematic Reviews and Meta-Analyses (PRISMA) [47] using the protocol developed by Crockett et al. [48] (with modifications) and the Cochrane guidance [49]. Included articles were published in the English language and among U.S. adults (>18 years old) from 1950 to 2014. Studies including a minority of individuals 15 to 18 years old [50,51] were retained to maintain sample size and power in the analysis, however, the effect of their inclusion was evaluated by completing the meta-analyses with and without these participants. Types of interventions included comparisons of nutrition labeling strategies at the point-of-selection in full-service restaurants, fast-food restaurants, or simulated settings. Only studies evaluating the implementation of quantitative calorie information for all or most menu items at point of purchase were included. Studies with multiple interventions (education; taxation; distinct labeling format: traffic light, healthy symbol, exercise equivalents, etc.) were included if the independent effect of quantitative nutrition labeling could be assessed. Besides calories and nutrient values, the succinct statement “2000 calories a day is used for general nutrition advice, but calorie needs vary” was not considered an educational component, but a regular labeling component according to the FDA requirement for this statement on menu labels. Groups within studies that included education, taxation, or/and a labeling format other than quantitative calorie/nutrient labeling were excluded. Outcome measures evaluated were estimates of energy, carbohydrates, total fat, saturated fat, and sodium purchased or consumed. There were no exclusion criteria based on study participants’ gender, body mass index, race, ethnicity, and social or economic status. 

Study designs eligible for consideration and criteria for inclusion were developed based on The Cochrane Consumers and Communication Review Group (CC&CRG) [49] supplementary guidance for review authors on study design, study quality, and analysis (Table 1). 

Quality of the studies was classified as “A” (Randomized Controlled Trial (RCT), quasi-RCT, Controlled Before and After (CBA) studies, and Interrupted Time Series (ITS) when they adhered to all criteria listed in Table 1) following the recommendation for inclusion in meta-analysis by CC&CRG and were used in an initial meta-analysis. Since few reports were found under this classification, a second meta-analysis was conducted broadening inclusion criteria to reports with study quality classified as “B”. In this classification, cross-sectional studies following all criteria as well as RCT, quasi-RCT, CBA/Before and After (BA) studies, and ITS following all but one of the criteria stated in Table 1 were allowed for inclusion (BA without a control group following all other criteria were also allowed for inclusion). Cross-sectional studies that did not meet one or more criteria and all other study designs that did not fill two or more criteria were excluded from this meta-analysis. After study design classification and selection, the Cochrane Collaboration’s tool for assessing risk of bias was applied to further identify and exclude studies containing high risk of bias [52]. The rationale for these stringent criteria was the intent to perform a meta-analysis including only strong study designs in order to produce the highest quality evidence. 

**Figure 1:**
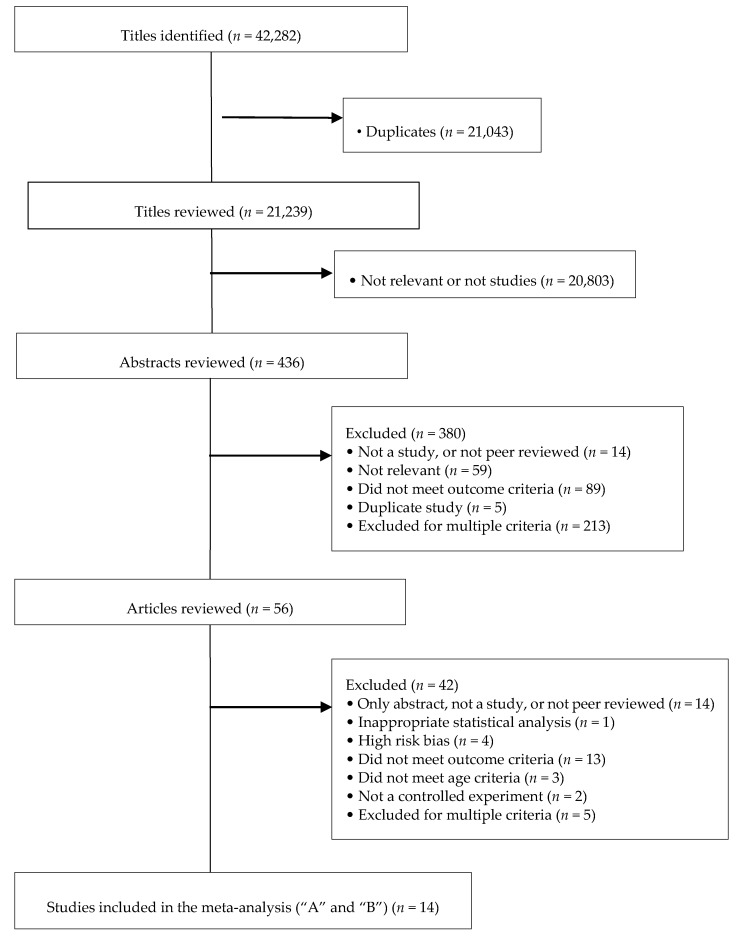
Flow diagram of the literature search and filtering results following the Preferred Reporting Items for Systematic Reviews and Meta-analyses (PRISMA) template.

Various databases were searched from January 2015 to March 2015 including: PubMed; Cumulated Index to Nursing and Allied Health Literature (CINAHL); Trials Register of Promoting Health Interventions (TRoPHI); The Cochrane Library; Food Science and Technology Abstracts; PsycINFO; Sociological Abstracts; ABI Inform (Proquest); Scopus database; Web of Science (Science Citation Index and Social Science Citation Index); Business Source Premier; EconLit; ProQuest Research Library; Hospitality and Tourism Complete. The following search strategy was used for the PubMed database: (1) “food labeling” or “food labelling” or “food label” or “food labels” or “point of sale” or “point-of-sale” or “point of sales” or “point-of-sales” or “point of purchase” or “point-of-purchase”; (2) (menu or nutrition or nutritional or nutritious or nutrient or nutrients or food or calorie or calories or caloric) and (symbol or symbols or label or labels or labeling or labelling or information or sign or signs or signage or content or contents or tag or tags or ticket or tickets)/ab,ti (abstract, title); (3) menu and (labels or labeling or labelling or label or education); (4) restaurant or restaurants or “fast food” or “food service” or “food services” or dine or dining or diner or diners or cafe or cafeteria or cafes or cafeterias/ab,ti; (5) ((food or nutrition or restaurant or “fast food”) and (policy or policies or legislation or law)); (6) (diet or diets or dietary or dietitian or dietetics or food or consumption or consumer or consumes or consumed or consume or consumers or meal or meals) and (habit or habits or habitual or prefer or preference or preferences or choice or choices or behavior or behaviors or behaviour or behaviours or behavioral or behavioural or plan or plans or planning or “choice behavior” or “decision making”); (7) 1 or 2 or 3; (8) 4 and 7; (9) 5 and 6; (10) 5 and 7; (11) 8 or 9 or 10. A similar search strategy was adapted and used for other databases. The PRISMA flow diagram of the literature search is outlined in Figure 1.

Two reviewers independently and sequentially assessed titles, abstracts, then papers identified in the search according to the inclusion and exclusion criteria outlined in the protocol. After each stage of assessment, the reviewers compared results and discussed any disagreements. If the disagreement could not be resolved, a third party was consulted to reach a final decision regarding inclusion. Data were collected using abstraction forms based upon the Cochrane Public Health Group data extraction template [53]. Once data were collected, the last author reviewed and verified the extracted information against that presented in the respective articles. All references and associated databases were maintained in EndNote X5 (Clarivate Analytics, Philadelphia, PA, USA) and references and corresponding results of assessment at each stage were maintained in Microsoft Excel (Microsoft, Redmond, WA, USA).

### 2.2. Data Collection

Data collected during abstraction included study setting, sample size, participant characteristics, study design, data collection methods, type of intervention/point of purchase labeling information, presence of interventions other than nutritional labeling (nutrition education, taxation, etc.), labeling format (quantitative vs. qualitative), and measurement of calories and nutrients purchased or consumed. The scope of the current study was limited to food ordering and consumption behaviors in restaurants and similar settings on a population or individual level. Food orders were reported as the total calories ordered or the difference in calories ordered between the intervention and comparison groups. Food consumption was reported as the “amount of food consumed or calories consumed as part of a meal” [48]. Besides ordered/consumed calories (kcal), studies including data for total fat (g), total carbohydrate (g), saturated fat (g), and sodium (mg) ordered/consumed were also evaluated regarding the possible effects of labeling on the selection and consumption of these nutrients. 

### 2.3. Statistics

Meta-analyses were performed in 2016 and applied the techniques found in Egger et al. [54] and Hedges et al. [55]. Meta-analysis outcomes can be derived with a variety of parameters, including effect sizes, confidence intervals, and standard deviations. For this particular meta-analysis, all parameters were converted to means and standard deviations, as the majority of reviewed manuscripts used this parameter in their results. If confidence intervals or standard errors were used in the articles, the appropriate formulas were used to derive standard deviations. For studies with multiple locations, pooled standard deviations were derived from the sites and means were averaged across sites in order to determine the pre- and post-time point parameters.

Once means and standard deviations were derived for each contributing study, the change in calories was calculated, along with the standard deviation of the difference. These in turn were used to derive the variance of the difference, which was used to find the pooled standard deviation. Study level weighted mean differences were calculated, and then used with the other outcomes to derive the overall meta-analysis weighted mean difference. Confidence interval error terms for the final weighted mean difference were calculated based on pooled standard deviation, total sample size, and the 97.5th percentile of the z-table in order to find the 95% confidence interval of the meta-analysis’ weighted mean difference. *Q* and *I*^2^ statistics were performed to test for heterogeneity [18] and data consistency. A random effect model was used to pool estimates across studies. Tree charts were produced using SAS v9.4 (SAS Institute, Cary, NC, USA) to visually show each study’s outcome and the overall meta-analysis outcome. Similar analyses were performed after stratification of studies in settings types (laboratory and non-laboratory or away-from-home settings) and for studies including each additional nutrient evaluated (carbohydrates, total fat, saturated fat, and sodium). 

## 3. Results

### 3.1. Selected Studies and Characteristics

Overall, 42,282 studies were identified via database searching (Figure 1). After eliminating duplicates, 21,239 studies were included in the title reading stage and 436 studies were retained for abstract evaluation. Fifty-six full-text publications were included in the full-text review and 42 were excluded [36,56,57,58,59,60,61,62,63,64,65,66,67,68,69,70,71,72,73,74,75,76,77,78,79,80,81,82,83,84,85,86,87,88,89,90,91,92,93,94,95,96]. Five studies [51,97,98,99,100] classified as “A” and nine [50,101,102,103,104,105,106,107,108] studies classified as “B” containing consumed and/or ordered calories as an outcome were included in the meta-analysis, collectively representing 0.07% of the total titles (without duplicates) identified (Figure 1). The sum of participants in all fourteen selected studies (“A” and “B”) was 552,029. Regarding study design, Roberto et al. [99] was a RCT; Temple et al. (2011) [108], Temple et al. (2010) [100], and Harnack et al. [51] were quasi-RCTs; Nelson et al. [104] was an ITS study; Elbel et al. [97], Finkelstein et al. [98], Krieger et al. [103], Pulos and Leng [106], Tandon et al. [107], Platkin et al. [105], and Downs et al. [102] were CBA or BA; and Auchincloss et al. [101] and Brissette et al. [50] were cross-sectional studies. Before-intervention means and standard deviations from studies with BA and ITS designs were included with the control means and standard deviations from the other study designs. Nine articles reported menu label implementation in away-from-home settings [50,97,98,101,102,103,104,106,107] and five involved laboratory settings [51,99,100,105,108] (Table 2).

### 3.2. Impact of Menu Labeling on Calories Ordered or Consumed

**Figure 2:**
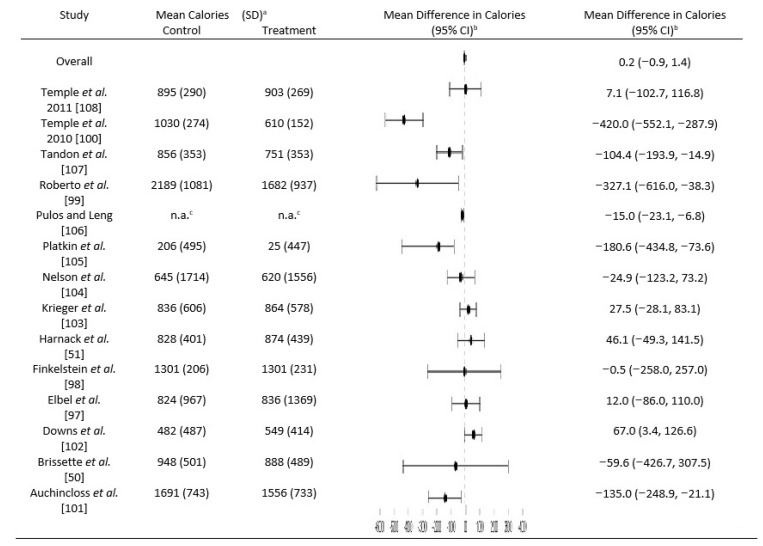
Forest plot showing the overall and study-specific mean differences with 95% CI. For each condition or subgroup, the full dot represents the point estimate of the menu label effect. The horizontal lines join the lower and upper limits of the 95% CI of these effects. Before-intervention mean calories are shown in the control mean calories column for before and after study designs used by Krieger et al. [103], Pulos and Leng [106], and Downs et al. [102]. ^a^ SD, Standard Deviation; ^b^ CI, Confidence Interval; ^c^ n.a.: Data not available.

The studies by Temple et al. (2011) [108] and Temple et al. (2010) [100] reported calories consumed only, while Nelson et al. [104], Elbel et al. [97], Finkelstein et al. [98], Krieger et al. [103], Pulos and Leng [106], Tandon et al. [107], Platkin et al. [105], Downs et al. [102], Auchincloss et al. [101], and Brissette et al. [50] reported calories ordered only. Roberto et al. [99] and Harnack et al. [51] looked at the effect of menu labeling on both calories ordered and consumed, but only mean ordered calories were used in this meta-analysis (Table 2) in order to maintain the most consistency with the other studies and avoid duplication. Results supported the analysis of no difference among calories chosen or consumed. Meta-analysis restricted to articles classified as “A” resulted in no statistically significant association (−0.13 calories; 95% CI: −0.83, 0.56). Moreover, results of the meta-analysis of all fourteen articles (“A” plus “B”) also indicated no statistically significant association between calorie labeling and lower caloric orders/consumption (*p* < 0.05), with an overall mean difference (95% CI) of −0.21 calories (95% CI: −1.36, 0.94) between pre- and post-treatment periods (Figure 2). Despite a focus on adults, Harnack et al. [51] and Brissette et al. [50] also included some individuals under 18 years old. However, the exclusion of these studies did not significantly change the final outcome (−0.19 calories; 95% CI: −1.25, 0.87).

Notably, the Forest plot indicates that most of the studies with an association between calorie labeling and lower caloric orders/consumption were conducted in laboratory settings (Figure 2).

Thus, a meta-analysis including only the studies conducted in laboratory settings was performed. A significant decrease in the post-period with a mean of −115.2 calories from pre-period (95% CI: −99.50, −130.87) was found. No significant difference was observed between pre- and post-period in natural, away-from-home settings only (0.03 calories; 95% CI: −0.96, 0.89). The results supported the hypothesis that the setting makes a difference to the efficacy of menu labeling in altering caloric choice or intake. High heterogeneity in the study designs and sample populations justified a need to adjust for these differences using random effects models. Yet, even after random effects modeling, the heterogeneity measures, *Q* and *I*^2^ statistics, remained significant (*p* < 0.05) in all meta-analyses conducted, indicating inherent heterogeneity in the studies and populations and/or outcomes that could not be controlled for. 

### 3.3. Impact of Menu Labeling on Carbohydrates, Fat, Saturated Fat, and Sodium Ordered or Consumed

Among the fourteen included studies in this meta-analysis, three also reported total ordered carbohydrate [51,101,106], fat [51,104,106], and saturated fat [51,97,101] and four reported ordered sodium [97,101,104,106] (Table 2). Harnack et al. [51] and Elbel et al. [97] used menu labels containing only calorie information; carbohydrate, saturated fat, and total fat content of foods chosen or consumed in Harnack et al. and saturated fat, sodium, and sugar for Elbel et al. were exclusively used for analysis purposes. Menu labeling in Auchincloss et al. [101], Nelson et al. [104], and Pulos and Leng [106] included the respective nutrients that were also analyzed as outcomes in Table 3. The meta-analysis of mean differences did not support an association between labeling and ordered carbohydrate (*p* < 0.05) (−0.1 g; 95% CI: −0.6, 0.5), fat (<0.1 g; 95% CI: −0.0, 0.0), saturated fat (<0.01 g 95% CI: −0.1; 0.1), and sodium (−0.6 mg; 95% CI: −3.7, 2.5) (Table 3), nor the hypothesis that intake of these nutrients would be altered as a result of menu labeling. 

## 4. Discussion

The results of this meta-analysis showed no effect of menu labeling on calories chosen, either ordered or consumed, among U.S. adults in natural settings, thus supporting the hypothesis and previous findings. A significant difference of −115.2 calories was found when meta-analysis was restricted to studies conducted in laboratory settings. This is a minimal calorie value in the context of a daily and usual dietary intake but demonstrates how the setting may influence efficacy of this intervention. Novel meta-analysis of carbohydrates, total fat, saturated fat, and sodium resulted in no significant effect of menu labeling on choosing or consuming these nutrients in food away from home among U.S. adults. Menu labeling was expected to result in alterations in choice or intake of these nutrients on the basis of efficacy to improve dietary choice or intake that had previously been undetected in meta-analyses results of null or minimal changes to caloric choice and intake. The lack of significant nutrient changes, however, are tempered with acknowledgement of the few studies included in those analysis.

A laboratory setting is not the setting that menu labeling is intended for as a real-life intervention. The controlled and manipulated laboratory setting may likely influence the behavior of participants [51]. For example, hunger may be differentially related to eating behavior in laboratory and real-life settings and this could lead to differences in caloric ordering/consumption patterns. Three of the five lab-based studies in this meta-analysis [99,100,108] quantified and controlled for hunger in the analysis, but none of the real-life setting studies measured this quality. Thus, knowledge of how this factor may have influenced results among the diverse settings is not known, and thus future research is needed. Other variables that may influence an individual’s choice in an external environment may be not easily translated into a laboratory setting. Along with the nature and extent to which one’s actions are scrutinized by others, the particular context and process by which a decision is embedded, and the self-selection of the individuals making the decisions, may be very different and influential in food and beverages ordered or consumed in real world vs. laboratory settings [109].

Previous studies indicate that taste [110,111], cost [110,111], accessibility [111], and convenience [111] are factors of higher relevance to patrons than nutritional concerns [110]. These variants may not be observed in laboratory experiments where food options are often limited, the food is offered for free, and the influence of accessibility and convenience cannot be evaluated. Mohr et al. [112] also identified a relative indifference to health consequences of behavior as one of the predictors of more frequent consumption of fast foods [112]. However, the sample of participants who agree to participate in laboratory setting studies may not represent a group with a similar attitude and set of beliefs regarding health in such a setting. Thus, factors not related to nutritional labeling alone are likely to be influencing the decision of food ordering/consumption in natural settings compared with laboratory settings, limiting the usefulness of laboratory controlled study findings to real-world applications. The challenge of evaluating the impact of nutrition labeling to food ordering/consumption in natural setting studies may be inherent to the limited ability of investigators to identify and quantify the variant and perhaps multiple factors that control a particular behavior. 

Previous meta-analyses and systematic reviews showed similar results of little to no difference in calories purchased/consumed due to menu labeling. Long el al. [42] found that labeling was associated with a –18.13 ordered kilocalorie reduction per meal, however, when only studies with control groups in restaurant settings were included, no significant association was found. Similarly, Swartz et al. [44] and Sinclair at al. [43] found no correlation between purchased/consumed calories and menu labeling. Notably, previous meta-analyses included studies with online ordering, non-restaurant/restaurant-like setting (i.e., coffee-shops) and hypothetical ordering [42], diverse age groups [42,43,44], and studies conducted in countries other than the U.S. [43]. These kinds of studies were excluded in our meta-analysis. We also included only studies adherent to stringent criteria based on CC&CRG guidance and classified as “A” and “B” (Table 1) that included quantitative calorie/nutrient labeling. Despite these restrictions, high heterogeneity was still present. The high heterogeneity may be due to the diversity of study designs (RCTs, quasi-RTCs, BA studies, ITS studies, and cross-sectional studies), varying dining settings (fast-food restaurants, laboratory settings, and full-service restaurants), and different outcome measures (calories ordered or consumed). This diversity highlights a need for more studies in real-world settings that use standardized, criteria-specified, and bound study designs and methodologies. 

Despite the finding of no effect of menu labeling on calories ordered/consumed in natural settings, menu labeling may have other impacts. Some evidence suggests that companies may be reformulating products (including decreasing calories content) in response to menu labeling regulations [113]. Pulos and Leng [106] reported that after seeing the results of their menu analyses, some locally owned restaurants modified portion sizes or ingredients. Some of the studies included in this meta-analysis also indicated that not all consumers or participants were aware of calorie labels [103,106]. Moreover, the way that calorie information was presented was not homogeneous, clearly reported, nor described at all in some of the included studies. Among studies providing clear and full description, calorie information was placed on the menu next to food item descriptions and price in three studies [51,99,101] and given as a separate pamphlet with the restaurant menu in Nelson et al. [104]. The optimal method for delivering nutrition information to the consumer (i.e., font-size, color, location, etc.) is yet to be determined. Educational materials to increase awareness and explain labeling use may inform food choice and enhance labeling use [51]. Previous nutrition knowledge was not assessed in most of the included studies, with the exception of the study by Brissette et al. [50], which found that knowledge of the calorie Recommended Dietary Allowance was not associated with the amount of purchased calories, yet the effect of nutrition knowledge on eating behavior may manifest in other ways and is another area for future hypotheses. Other factors, as mentioned above, may also influence calories ordered and/or consumed, such as consumer preference for taste, convenience, or price. For example, taxation decreased the purchasing of more calorically dense foods in obese, but not non-obese participants in Temple et al. (2011) [108], results that were not included in this meta-analysis, indicating that specific participant characteristics may differentially be influenced by such manipulations. Indirect impacts of menu labeling may also result; for example, menu labeling policies may lead to a shift in broader social norms to adopt healthier diets over time. Thus, menu labeling may have a potential to change dietary behavior beyond the results of this meta-analysis and other previous studies.

Consideration of dietary intake beyond calories, such as the inclusion of nutrient-dense foods and beverages—vegetables, fruits, whole grains, fat-free or low-fat milk and milk products, seafood, lean meats and poultry, eggs, beans and peas, and nuts and seeds and limiting simple carbohydrates, fat, saturated fat, and sodium is critical to understanding the efficacy of menu labeling to support dietary changes that prevent diet-related chronic diseases like diabetes, dyslipidemia, and hypertension. [114,115,116,117]. Evaluation of nutrients provides evidence of potential changes in the type or quality of nutrients ordered/consumed and not only the quantity of nutrients ordered/consumed due to menu labeling changes. In our meta-analysis, significant differences in the calories ordered or consumed for carbohydrates, fat, saturated fat, and sodium were not observed after menu labeling implementation. Analysis of nutrients purchased or consumed in menu labeling interventions is of growing interest, but still few studies have included evaluation of the impact of menu labeling on the ordering/consumption behavior of nutrients. Thus, despite our results, future studies should continue to include evaluation of menu labeling on nutrients ordered/consumed to provide more robust insights into these broader aspects of menu labeling outcomes. Future studies should consider evaluating additional nutrients or dietary components such as added sugars, dietary fiber, calcium, vitamin D, potassium, and overall indicators of dietary quality such as the Healthy Eating Index in order to provide a more complete picture of dietary intake.

This meta-analysis included only studies adherent to stringent criteria, based on CC&CRG guidance and classified as “A” and “B” study designs, and to our knowledge is the first to evaluate the effect of menu labeling on calories and carbohydrates, fat, saturated fat, and sodium ordering/intake. Because few articles were found to contain evaluation of those nutrients, these results highlight the need of nutrient evaluation in future studies to draw more robust conclusions. Investigation of menu labeling effects separately for laboratory and natural settings allowed results specific to real-life environments. 

Although only stringent criteria were used to grade study designs for inclusion, the diversity in study methodologies resulted in high heterogeneity. This meta-analysis did not assess what effect the posting of daily calories/nutrient requirements in addition to menu labeling had on calories and nutrients consumed and ordered. Consumer knowledge of nutrition daily requirements may affect the choice of food consumed away from home and how these choices are balanced with other food intake throughout the day. U.S. adults were included in this analysis, but specific populations, such as obese individuals or children, may react to menu-labeling differently. Finally, there exists the possibility that other unpublished studies were not included in this meta-analysis. 

## 5. Conclusions

Menu labeling had no effect on calories, carbohydrates, fat (total and saturated), and sodium ordered and consumed away from home among U.S. adults in natural settings. This meta-analysis, to our knowledge, was the first to include nutrients ordered/consumed along with calories in response to restaurant menu labeling. As menu labeling is implemented across the U.S., further examination of the long-term impacts on the full spectrum of dietary intake will inform the use of this policy intended to support the prevention and reduction of obesity and other chronic diseases.

## Figures and Tables

**Table 1:** Study designs eligible for consideration and criteria for inclusion.

Study Design ^a^	Criteria	Study Classification	
		All Criteria Fulfilled	All but ≤1 Criteria Fulfilled
Randomized Controlled Trial (RCT) ^b^	• Unique groups of participants in experimental and controlled conditions (not crossed over)	A	B
Quasi-Randomized Controlled Trial (Quasi-RCT)	• Unique groups of participants in experimental and controlled conditions (not crossed over)	A	B
Controlled Before-and-After (CBA) study	At least two intervention sites and two control sites A control group should be used for comparison The timing of the periods for study for the control and intervention groups should be comparable The intervention and control groups should be comparable on key characteristics	A	B ^c^
Interrupted Time Series (ITS) Study	A clearly defined point in time at which the intervention occurred Collection of at least three data points before and three data points after the intervention was introduced	A	B
Cross-Sectional Study	At least two intervention sites and two control sites A control group should be used for comparison	B	Exclude

Abbreviations: RCT, Randomized Controlled Trial; CBA, Controlled Before and After; ITS, Interrupted Time Series. ^a^ Study designs were identified based on the Cochrane Consumers and Communication Review Group study design guide [49]; ^b^ RCT designation was used only when allocation was clearly not decided by the clinician or the participant, and that assignment to one group or another was not predictable; ^c^ Before and after studies without a control group adhering to other criteria were also included in this group.

**Table 2:** Main characteristics of included studies.

Study	Sample Characteristics				Study Design **	Study Design Classification	Setting ***	Meal Type	Outcomes of Interest Assessed ****
	*N* *	Unit of Analysis	Age (Years)	Other					
Auchincloss et al., 2013 [101]	648	Number of participants	>18	60% female; 50% black/African American; 15 years mean education; 40–50% overweight; >41% income over $60,000	Cross-sectional	B	Full-service restaurants ^f^	Dinner	Ordered calories; total carbohydrate; saturated fat; sodium
Brissette et al., 2013 [50]	1094	Number of participants	>15	71% non-Hispanic white; 59% male; 42% ≤ high school education;	Cross-sectional	B	Fast-food restaurants	Lunch (92%) and dinner (8%)	Ordered calories
Downs et al., 2013 [102]	1094	Number of participants	>18	36% African American, 53% female; 49% had a BMI > 25 kg/m^2^	BA (not controlled)	B	Fast-food restaurants	Lunch	Ordered calories
Elbel et al., 2009 [97]	1156	Number of participants	>18	66% black; 38% male; almost 50% with ≤ high school diploma; low income community	CBA	A	Fast-food restaurants	Lunch and dinner	Ordered calories; saturated fat; sodium
Finkelstein et al., 2011 [98]	540,552	Number of transactions	n.a.	King County and adjacent county Taco Time Northwest customers in Washington, U.S.	CBA	A	Fast-food restaurants	Various meals	Ordered calories
Harnack et al., 2008 [51]	301 ^a^	Number of participants	>16	About 75% white; 61% female; 37% with some college education; 56% had a BMI > 25 kg/m^2^	Quasi-RCT	A	Laboratory setting	Dinner	Ordered and consumed calories; total carbohydrate; total fat; saturated fat
Krieger et al., 2013 [103]	2746 ^b^	Number of participants	>40	76% non-Hispanic white; 59% male; 32% from a low income/diverse area	BA (not controlled)	B	Fast-food restaurants	Lunch	Ordered calories
Nelson et al., 1996 [104]	3234	Number of purchased entrees	18–81	>53% male; Recruited from University restaurant, >60% with master’s degree or higher	ITS (Only four data collection points)	B	Full-service restaurant ^g^	Lunch	Ordered calories, fat and sodium (calculated from Appendix B in Nelson et al.)
Platkin et al., 2014 [105]	104 ^c^	Number of participants	18–34	71% black or Hispanic; female; recruited on college campus; mean BMI 28 kg/m^2^	BA (single-location)	B	Laboratory setting	Lunch	Ordered calories
Pulos and Leng, 2010 [106]	206	Number of purchased entrees	>18	55% female	BA (not controlled)	B	Full-service restaurant ^h^	Lunch and Dinner	Ordered calories, total carbohydrate, total fat, sodium
Roberto et al., 2010 [99]	293	Number of participants	>18	50% female; 55% white; 85% with some college or higher; mean BMI 25 kg/m^2^	RCT	A	Laboratory setting	Dinner	Ordered and consumed calories
Tandon et al., 2011 [107]	242 ^d^	Number of participants	n.a.	80% females; 70% college degree or higher; 39–70% with income >$90,000; 64% with a BMI > 25 kg/m^2^	BA (single-location)	B	Fast-food restaurant	Various meals	Ordered calories
Temple et al., 2010 [100]	47	Number of participants	18–50	51% female; 91% completed some college or higher; 43% with income <$10,000; mean BMI 26 kg/m^2^	Quasi-RCT	A	Laboratory setting	Lunch	Consumed calories
Temple et al., 2011 [108]	102 ^e^	Number of participants	Adults	55% female recruited from University; 47% white; 51% with income <$9999; 53% completed some college; 68% had a BMI > 25 kg/m^2^	Quasi-RCT (crossed-over)	B	Laboratory setting	Lunch	Consumed calories

Abbreviations: BA, Before and After; BMI, Body Mas Index; CBA, Controlled Before and After; n.a., Data not available; RCT, Randomized Controlled Trial; ITS, Interrupted Time Series. * After applicable exclusions, as follows: ^a^ Groups “calories plus price” and “price interventions” were excluded from this analysis; ^b^ Adults ≥18 were included in this meta-analysis so participants stratified to the ≥14 and <40 year group in Krieger et al. were excluded along with those from coffee chains; characteristics shown reflect those of the entire sample; ^c^ The group receiving calorie plus exercise equivalents in lunch Section 2 was excluded from this analysis; ^d^ Only data from parents were included in this analysis; ^e^ Only data from participants in experimental condition 1 were included in this analysis. Moreover, the group receiving traffic light labeling treatment was excluded from this analysis. ** The general study designs follow criteria stated in Table 1, unmatched criteria are presented in parenthesis. *** Additional nutrient menu labeling was provided as follows: ^f^ sodium, saturated fat, trans fat, carbohydrates; ^g^ fat, cholesterol, sodium, dietary fiber; ^h^ fat, sodium, carbohydrates. **** Other nutrients were evaluated in the studies of Auchincloss et al. [101] (trans fat); Elbel et al. [97] (sugar); Pulos and Leng (carbohydrates) [106]; Harnack et al. [51] (calcium, vitamin C, dietary fiber, and protein) and Nelson et al. [104] (dietary fiber) but were not included in this meta-analysis because there were too few studies evaluating these outcomes.

**Table 3:** Impact of menu labeling on carbohydrates, fat, saturated fat, and sodium ordered or consumed.

Nutrient	Mean (SD)		Mean Difference (95% CI)
	Control	Treatment	
**Total Carbohydrates (g)**			
Harnack et al., 2008 [51]	105.7 (39.2)	110.3 (63.3)	4.6 (−7.3, 16.5)
Auchincloss et al., 2013 [101]	131 (72)	115 (64)	−16 (−26.5, −5.5) *
Pulos and Leng, 2010 [106]	n.a. ^a^	n.a.	−0.2 (−1.1, 0.8)
Overall			−0.1 (−0.6, 0.5)
**Total Fat (g)**			
Harnack et al., 2008 [51]	32.5 (18.6)	34.3 (19.3)	1.8 (−2.5, 6.1)
Nelson et al., 1996 [104]	36.8 (10.5)	34.4 (8.9)	−2.4 (−3.1, −1.8) *
Pulos and Leng, 2010 [106]	n.a.	n.a.	−1.6 (−2.2, −1.0) *
Overall			<0.1 (−0.0, 0.0)
**Saturated Fat (g)**			
Harnack et al., 2008 [51]	9.7 (6.7)	10.7 (7.6)	1.0 (−0.6, 2.6)
Auchincloss et al., 2013 [101] ^b^	36.5 (23.9)	33.5 (22.1)	−3.0 (−6.5, 0.5)
Elbel et al., 2009 [97]	11.8 (19.7)	11.4 (19.7)	−0.4 (−2.0, 1.2)
Overall			<0.1 (−0.1, 0.1)
**Sodium (mg)**			
Auchincloss et al., 2013 [101] ^b^	3315 (1389)	3111 (1460)	−204 (−423.8, 15.8)
Nelson et al., 1996 [104]	2077 (1032)	2113 (1045)	36 (−34, 106)
Pulos and Leng, 2010 [106]	n.a.	n.a.	−45.7 (−74.5, −16.8) *
Elbel et al., 2009 [97]	1392 (1925)	1476 (2396)	84 (−96, 264)
Overall			−0.6 (−3.7, 2.5)

* indicates significant mean differences between control and treatment conditions for the nutrient assessed. Abbreviations: SD, Standard Deviation; CI, Confidence Interval; g, grams; mg, milligrams. ^a^ Data not available, mean differences and CI were used in meta-analysis; ^b^ Significant difference was determined in Auchincloss et al. but not in this meta-analysis most likely due to differing analytical methods of comparison.
